# Neurofibromatosis 2: hearing restoration options

**DOI:** 10.5935/1808-8694.20120020

**Published:** 2015-11-20

**Authors:** Tatiana Alves Monteiro, Maria Valeria Schmidt Goffi-Gomez, Robinson Koji Tsuji, Marcos Queiroz Telas Gomes, Rubens Vuono Brito Neto, Ricardo Ferreira Bento

**Affiliations:** a ENT Physician; Ear Surgery and Lateral Skull Base Surgery Fellow - Department of Otorhinolaryngology - Medical School of the University of São Paulo - HCFMUSP; b PhD; Speech and Hearing Therapist - Department of Otorhinolaryngology - HCFMUSP; c PhD. Assistant Physician - Department of Otorhinolaryngology - Medical School of the University of São Paulo - HCFMUSP; d Neurosurgeon; Assistant Physician - Department of Neurosurgery - HCFMUSP; e Senior Associate Professor - Department of Otorhinolaryngology - Medical School of the University of São Paulo - HCFMUSP; f Full Professor - Department of Otorhinolaryngology - Medical School of the University of São Paulo - HCFMUSP. Departamento de Otorrinolaringologia e Neurocirurgia do Hospital das Clinicas da Faculdade de Medicina da Universidade de São Paulo

**Keywords:** auditory brainstem implants, cochlear implants, deafness, neurofibromatosis 2

## Abstract

Neurofibromatosis 2 (NF2) is an autosomal dominant disease in which hearing loss is predominant. Auditory restoration is possible using cochlear implants (CI) or auditory brainstem implant (ABI).

**Objective:**

To assess the auditory results of CI and ABI in NF2 patients and review the literature.

**Methods:**

Four NF2 patients were prospectively evaluated. They were submitted to tumor resection followed by ipsilateral CI or ABI depending on cochlear nerve preservation. Long term auditory results were described for CI (12 months) and ABI (48 months).

**Results:**

All patients achieved auditory perception improvements in their hearing thresholds. The CI patient does not recognize vowels or sentences. The 3 ABI patients discriminate 70% of vowels and 86% in the 4-choice test. One of them does not recognize sentences. The other two recognize 100% of closed sentences and 10% and 20% of open sentences.

**Conclusion:**

The choice of implant type to restore hearing to NF2 patients will relay on anatomical and functional cochlear nerve preservation during tumor resection surgery. Although our experience was different, the literature shows that if this condition is achieved, CI will offer better auditory results. If not, ABI is recommended.

## INTRODUCTION

Neurofibromatosis type 2 (NF2) is a dominant autosomal disease which affects 1:40,000 individuals^1^^,^^2^. In these cases there is a two allele mutation on the chromosome 22 long arm tumor suppressor gene^1^^,^^2^. The patients usually develop multiple schwannomas along the spine and in the skull. Bilateral vestibular schwannoma happens in 90% of those with the gene^1^. These patients will invariably develop progressive sensorineural hearing loss, characterized by poor sound discrimination. Tinnitus and unbalance may also happen^1^.

The auditory rehabilitation of patients who evolved to a severe-profound hearing loss, until very recently was restricted to them learning lip reading. In 1979, House and Hitselberger started to change this story when they performed the first single channel auditory brainstem implant (ABI) in a NF2 deaf patient^3^. The implant electrode bundle has improved with the years, going from 8 to 21 electrodes^4^. This technological progress has benefited over 700 patients implanted with the ABI, with variable auditory responses^1^^,^^2^^,^^4^, ^5^, ^6^, ^7^, ^8^.

During the 90's, the cochlear implant (CI) also became available for the rehabilitation of deaf patients with NF2 submitted to tumor resection with cochlear nerve preservation^9^, ^10^, ^11^, ^12^, ^13^, ^14^, ^15^.

The auditory rehabilitation of NF2 patients may be carried out in two different ways (CI or ABI). We will, hereby, report on the auditory results from four patients with NF2 submitted to brainstem or cochlear auditory implant surgery in our clinic, and review the literature on this topic.

## METHOD

We prospectively assessed four NF2 patients submitted to vestibular schwannoma exeresis surgery during February of 2006 and March of 2009. The ipsilateral brainstem implant (ABI) or the cochlear implant (IC) was carried out in the same procedure, depending on cochlear nerve preservation.

Clinical and radiological data were collected before surgery, stressing auditory performance, auditory deprivation time and tumor size.

Three patients were implanted through the translabyrinthine approach with the Nucleus 24® ABI (Cochlear Corporation) after tumor removal. In order to assess the lateral wall of the IV ventricle (Luschka's foramen). As anatomical landmarks, we used the following: the IX cranial nerve, the acoustic-facial trunk, the cerebellar flocculus and the choroid plexus. During the surgeries, the VII, IX, X and XI ipsilateral cranial nerves were continuously monitored (NIM Response-2; Medtronic Xomed).

Brainstem electrically evoked auditory potentials (electrical ABR) were used to check for proper positioning of the electrode bundle, by means of the Biologic Navigator Pro (Bio-logic Systems Corp.®) device, coupled to the implant stimulation interface through a sync cable.

In one patient, the cochlear nerve anatomical preservation was possible during tumor exeresis through the retrolabyrinthine approach. The promontory electrical stimulation test during surgery showed a present response, although with poor morphology and dubious reproducibility. We decided to use the Nucleus Freedom Contour Advanced^®^ (Cochlear Corporation) simultaneous cochlear implant through the round window. In such case, intraoperative neural telemetry showed no response in the five electrodes tested. The electrode bundle positioning was checked after surgery through Stenvers and transorbital view x-rays.

Checking electrode impedances, both in ABI as well as in CI showed inadequate values.

ABI activation was done approximately 1 month after the surgery, with the patient awake in an ICU unit with cardiorespiratory monitoring. The electrodes were sequentially activated with a monopolar current, with a gradual increase for recording a comfortable auditory sensation and side effects. The speech coding strategy used in the three patients was the Spectral peak (Speak). CI activation was carried out after surgery and the speech coding strategy was the advanced combination encoders (ACE).

The auditory results after 48 months using the ABI and 12 months using the CI were expressed in: tonal thresholds in a sound field, four-choice test, vowel and phrases recognition index presented in a closed and open format, telephone use.

For the literature review, we used the following keywords: (cochlea or cochlear or auditory or ear) and (brain implant or brain implants), in the Medline, Lilacs and Scielo databases, with the limit of publication period up to March of 2010. We found 1118 publications. Of these, there were 17 clinical trials with NF2 patients submitted to cochlear or brainstem auditory implant, in English or Portuguese. Each study was classified according to its strength of evidence, according to the classification from the Oxford Centre for Evidence-based Medicine for studies on treatment (Table 1). Later on, these studies were also classified according to the degree of recommendation, nine publications with recommendation grade B and eight with recommendation grade C (Table 1).

**Table 1:** Classification of the papers used in the Literature Review.

Authors	Year	Sample	Type of Study	Intervention	Strength of Evidence	Recommendation Grade
Tono et al.	(1996)	1	Case Report	CI	4	C
Grahan et al.	(1999)	1	Case Report	CI	4	C
Erbinger et al.	(2000)	88	Clinical Trial	ABI	2b	B
Nevinson et al.	(2002)	27	Treatment Results Observation	ABI	2c	B
Nolle et al.	(2003)	1	Case Report	CI	4	C
Otto et al.	(2004)	20	Treatment Results Observation	ABI	2c	B
Kanowitz et al.	(2004)	18	Treatment Results Observation	ABI	2c	B
Colletti & Shannon	(2005)	20	Treatment Results Observation	ABI	2c	B
Aristegui & Denia	(2006)	1	Case Report	CI	4	C
Lustig et al.	(2006)	7	Treatment Results Observation	CI	2c	B
Behr et al.	(2007)	20	Treatment Results Observation	ABI	2c	B
Bento et al.	(2008)	4	Case Series prospective	ABI	4	C
Grayeli et al.	(2008)	31	Case Series retrospective	ABI	4	C
Otto et al.	(2008)	10	Treatment Results Observation	CI	2c	B
Vincenti et al.	(2008)	5	Cohort Study prospective	ABI/CI	2b	B
Maini et al.	(2009)	10	Case Series retrospective	ABI	4	C
Temple et al.	(2009)	1	Case Report	CI	4	C

ABI: Auditory Brain Stem Implant

CI Cochlear Implant

This study was approved by the Ethics Committee for Research Project Analysis of our institution, under protocol number 1135/07. Extensive explanation on the risks and benefits of brainstem auditory implant surgery and the option to continue only with the lip reading training therapy were given to the patients. Signing the Informed Consent Form meant agreeing to participate in the study.

## RESULTS

The three patients implanted with an ABI were males, with a mean age of 26.3 ± 1.5 years, mean deafness duration of 6.0 ± 4.0 years and mean tumor size of 3.2 ± 1.4 cm (Table 2). They all regularly used their devices for more than eight hours a day. None of them used a personal sound amplification device (PSAD) in the contralateral ear.

**Table 2:** Clinical and epidemiological data of the NF2 patients implanted with ABI and CI.

Deafness tumor	Age (years)	Gender	Duration (years)	Side	Size (cm)	Approach	Implant type
Case 1	28	M	10	L	3.5	TL	N24 ABI
Case 2	25	M	6	L	4.0	TL	N24 ABI
Case 3	26	M	2	L	2.0	TL	N24 ABI
Case 4	36	F	1	R	1.5	RL	CI N24 RE

TL: Translabyrinthine approach; RL: Retrolabyrinthine; N24 ABI: Auditory Brainstem Implant Nucleus 24; IC 24 RE: Cochlear Implant Freedom Contour; L: Left; R: Right.

No additional postoperative complication was observed on these patients by placing the ABI. Two patients had post-operative facial paralysis, keeping a House-Brackmann score of VI. One developed a CSF fistula, treated by an external lumbar shunt and compressive brain dressing. The adverse effects seen during the ABI activation were: throat stimulation (1), torso (2), upper (2) and lower limbs (1), nausea (1) and nystagmus (1). The number of electrodes which generate hearing sensation without side effects varied between 2 and 14 (Table 3).

**Table 3:** Number of active electrodes in patients implanted with the ABI and the CI.

# of active electrodes	3 m	12 m	36 m	48 m
Case 1	2	2	3	3
Case 2	6	5	4	4
Case 3	14	14	14	14
Case 4	5	11	x	x

x: does not apply by the duration of cochlear implant use.

**Graph 1:**
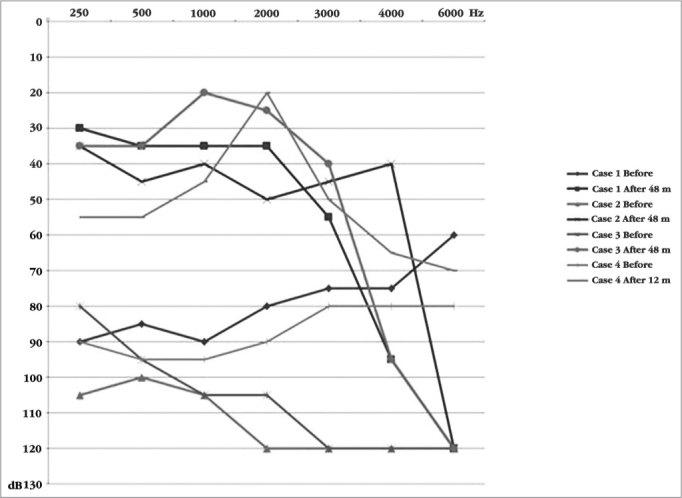
Free field audiometrie thresholds before and after the implant.

The mean value of the audiometric threshold in the free field using the device with ABI patients in the frequencies of 500; 1,000; 2,000 and 4,000 Hz was 36.7 ± 5.7 decibels (Graph 1). The tonal thresholds reached were similar among the patients and were maintained stable throughout the years. Auditory performance varied, one patient does not recognize open sentences and only 20% closed ones. The other two recognize 100% of the phrases and 10% and 20% open (Table 4). Only one of them was capable of communicating on the phone.

**Table 4:** Auditory results in patients implanted with the ABI and CI at 3, 12, 36 and 48 months after surgery.

	Vowels (%)			Four Choice (%)					Closed Phrases (%)				Open phrases (%)			
	3 m	12 m	36 m	48 m	3 m	12 m	36 m	48 m	3 m	12 m	36 m	48 m	3 m	12 m	36 m	48 m
Case	NR	NR	100	100	NR	100	100	100	NR	NR	100	100	NR	NR	20	20
Case 2	73	26	40	30	91	41	50	58	30	0	0	0	0	0 0	0	
Case 3	66	NR	20	80	100	100	100	100	100	100	100	100	0	0	60	60
Case 4	0	0	x	x	0	0	x	x	0	0	x	x	0	0	x	x

NR: not done; x: does not apply by the duration of cochlear implant use.

The patient implanted with CI was 36 years old, with a 10-year history of progressive hearing loss, evolving towards a severe hearing loss for 1 year (Table 2). The tumor on the implanted side measured 1.5 cm (Table 2). She also used her implant regularly for more than 8 hours a day and used a contralateral PSAD for 7 months, with low sounds perception.

Upon activation, there were five electrodes with auditory sensation and without side effects. After 12 months of stimulation, 11 electrodes could be activated without side effects. The audiometric threshold in a free field improved after the CI, the mean values for the following frequencies: 500; 1,000; 2,000 and 4,000 Hz was 46.2 decibels (Graph 1). Despite the improvement in tonal thresholds, the patient managed to detect only the presence of sound, not discriminating phrases, words, vowels, and rhythm (Table 4).

## SYSTEMATIC REVIEW OF THE LITERATURE AND DISCUSSION

The auditory rehabilitation of NF2 patients, who develop deafness, may be carried out in two ways: auditory cochlear or brainstem implants^2^^,^^3^. The choice of implant type to the utilized will depend on the anatomical and functional preservation of the cochlear nerve during the tumor resection surgery^2^^,^^3^. In these regards, electrophysiological tests have a crucial role in the identification of cochlear nerve responses.

This can be done during the procedure, studying the cochlear nerve action potential^2^. If this potential is present, the cochlear implant can be placed simultaneously with the tumor resection. Should this potential be absent or not reproducible, one should perform the promontory electrical stimulation test 6 to 8 weeks after the procedure^2^. In the immediate post-op, the electrical stimulation test on the promontory may be negative because of cochlear nerve neuropraxia, caused by surgical manipulation^2^. Such test is based on the introduction of a transtympanic needle, under local anesthesia, which will contact the promontory. The electrical stimulus is provided to the promontory in five different frequencies (50, 100, 200, 400 and 800 Hz) and the current level, initially of 0 μAmps, is progressively increased until the patient hears of perceives the stimulus^2^. The test is deemed positive when there is mild perception of a tone and differentiation between the different pitches. The test is deemed negative when the patient feels only a discomfort caused by the electrical stimulus^2^.

The literature has data on eight patients with anatomical preservation of the cochlear nerve, submitted to CI surgery, but without functional proof by an electrophysiological test, with results which were lower than those from a group of patients with cochlear nerve functional confirmation^15^^,^^16^ (Table 5). Only three of the eight cases had phrases discrimination^15^^,^^16^ (Table 5). The data from the other group with six patients with anatomical and functional preservation of the cochlear nerve, proven by electrophysiological tests, showed phrase discrimination in all the cases, with a mean value of 74.5 ± 18.8% (50-100)^2^,^16^,^17^ (Table 5).

**Table 5:** Auditory results in patients implanted with the CI described in the literature.

Case	Age (years)	Deafness duration (months)	Follow up time (months)	Open phrases (%)	Electrophysiological test
Vincenti et al. (2008)^2^	47	3	12	90	Good promontory stimulus
Vincenti et al. (2008)^2^	24	0	12	81	Good cochlear nerve action potential
Vincenti et al. (2008)^2^	32	3	12	50	Good promontory stimulus
Vincenti et al. (2008)^2^	36	3	12	0	Doubtful cochlear nerve potential and promontory stimulus
Tono et al. (1996)^11^	31	15	12	62	Good promontory stimulus
Temple et al. (1999)^17^	15	9	12	100	Good promontory stimulus
Nölle et al. (2003)^14^	16	24	24	88	Good promontory stimulus
Grahan et al. (1999)^12^	44	84	6	34	Promontory stimulus present
Aristegui & Denia (2005)^15^	52	ND	18	100	NCO (Obs. Viable contralateral hearing)
Lustig et al. (2006)^16^	35	5	28	0	NCO (Obs. Viable contralateral hearing)
Lustig et al. (2006)^16^	51	5	40	0	NCO (Obs. Viable contralateral hearing)
Lustig et al. (2006)^16^	16	13	30	0	NCO
Lustig et al. (2006)^16^	41	36	17	0	NCO
Lustig et al. (2006)^16^	28	4	88	0	NCO
Lustig et al. (2006)^16^	50	22	18	98	NCO
Lustig et al. (2006)^16^	57	96	9	21	NCO

ND: Not Described; NCO: Not Carried Out.

However, in one case of promontory stimulation, with a long time between the tumor resection surgery and the cochlear implant, the discrimination of open phrases was worse^12^ (Table 5). In this case, the cochlear basal turn was found to be ossified during surgery, suggesting that the insertion of the electrode bundle in the cochlea, when not together with the tumor removal, must be carried out in the shortest possible time in order to avoid cochlear ossification consequent to surgical manipulation^12^. In one case with cochlear nerve anatomical preservation, but with questionable response upon the promontory electrical stimulation test as to the nerve's action potential, speech discrimination was not achieved^2^ (Table 5). We found the same thing in our CI patient; despite cochlear nerve anatomical preservation, the promontory electrical stimulation test yielded poor responses and the patient did not discriminate the sounds; thus, the importance of functional proof of the nerve in order to obtain a good audiological result.

When it was not possible to spare the cochlear nerve during the vestibular schwannoma resection surgery or when its anatomical sparing did not generate electrophysiological responses, the only feasible option for the auditory rehabilitation is the brainstem auditory implant^1^^,^^2^^,^^4^, ^5^, ^6^^,^^16^^,^^18^, ^19^, ^20^, ^21^.

Studies have shown that the ABI (Auditory Brainstem Implant) has variable audiological responses. Nonetheless, it is a consensus that the ABI enables the perception of alert environmental sounds between 85%^7^ to 96.2%^5^ of the patients after one year of surgery, besides enhancing lip reading^5^^,^^18^^,^^20^.

Patients with auditory perception recognize the difference between the human speech sound and environmental sound and 64% recognize the difference between the voices from female/male and adult/children^5^. The mean open phrase recognition after one year using the ABI varies among the studies from 5% to 48%, together with lip reading, the values vary between 25 and 79%^20^. In general, the ABI provides an additional gain between 30% and 40% compared to lip reading only^1^^,^^2^^,^^5^^,^^18^, ^19^, ^20^. Using a telephone is not an expected result, being the exception to the rule^2^^,^^5^.

The benefit provided by the ABI does not discard training in lip reading and multidisciplinary rehabilitation is very important for patient improvement^1^. Even after one year using the ABI, improvements continue to be seen^4^. In our series, one of the patients had a worsening in his auditory perception along the years, despite the fact that his tonal thresholds had improved. Of the six working electrodes he had, only four remained. We do not know, for sure how to explain such worsening, but since this is the patient with the largest tumor, we speculate that there could have been a progressive damage to the cochlear core, corroborated by the progressive electrode deactivation time. The electrodes are deactivated when the stimuli do not generate significant auditory input or causes extra-auditory inputs (side effects by stimulation from adjacent cores). In this patient, as time passed, it was necessary to increase the electrical charge in order to generate an auditory input, which caused greater stimulation scatter and more pronounced side effects.

Comparing the literature results obtained from NF2 patients with cochlear nerve function and anatomical preservation, submitted to CI with those submitted to ABI, there were no great differences in relation to the recognition of vowels and consonants^2^. The recognition of dissyllable words and phrases was higher with the cochlear implant^2^. Moreover, most of the patients implanted with a CI are able to use the telephone.

## CONCLUSION

Patients with neurofibromatosis type 2 have now the technological resources which help in the auditory rehabilitation, providing an additional gain upon lip reading training. Although our experience has shown the opposite, literature shows that when possible to anatomically and functionally spare the cochlear nerve, the cochlear implant yields better auditory and language results. When cochlear nerve sparing is not possible, the brainstem auditory implant may be used for the same end.
